# Anima: Modular Workflow System for Comprehensive Image Data Analysis

**DOI:** 10.3389/fbioe.2014.00025

**Published:** 2014-07-30

**Authors:** Ville Rantanen, Miko Valori, Sampsa Hautaniemi

**Affiliations:** ^1^Research Programs Unit, Genome-Scale Biology and Institute of Biomedicine, Biochemistry and Developmental Biology, University of Helsinki, Helsinki, Finland

**Keywords:** quantification, automated analysis, image analysis, high-throughput, superplatform

## Abstract

Modern microscopes produce vast amounts of image data, and computational methods are needed to analyze and interpret these data. Furthermore, a single image analysis project may require tens or hundreds of analysis steps starting from data import and pre-processing to segmentation and statistical analysis; and ending with visualization and reporting. To manage such large-scale image data analysis projects, we present here a modular workflow system called Anima. Anima is designed for comprehensive and efficient image data analysis development, and it contains several features that are crucial in high-throughput image data analysis: programing language independence, batch processing, easily customized data processing, interoperability with other software via application programing interfaces, and advanced multivariate statistical analysis. The utility of Anima is shown with two case studies focusing on testing different algorithms developed in different imaging platforms and an automated prediction of alive/dead *C. elegans* worms by integrating several analysis environments. Anima is a fully open source and available with documentation at www.anduril.org/anima.

## 1 Introduction

Automated microscopes and image acquisition systems enable experiments that can easily produce millions of images in a single experiment (Tanasugarn et al., 1984; Conrad and Gerlich, 2010; Shamir et al., 2010). Manual analysis of such vast amount of image data is impossible, and a wide array of computational methods is needed to translate raw data files into biological and medical knowledge (Eliceiri et al., 2012). Accordingly, a number of image analysis tools, most notably CellProfiler (Carpenter et al., 2006), ImageJ (Schneider et al., 2012) and its extension Fiji (Schindelin et al., 2012), and several others as reviewed by Eliceiri et al. (2012), have been introduced. They have been used successfully especially in extracting intensity and shape features from various biological objects.

A key challenge with the existing methods is that their concurrent use is hindered by different input/output requirements. For example, it is challenging to add new algorithms to the analysts’ use as a new implementation needs to be programed (Cardona and Tomancak, 2012). Furthermore, existing software typically do not include options for high-order statistical or computational downstream analyses (Huang, 2010), and the use of various software without systematic framework leads easily to delays and errors. In order to overcome these issues, there is a need for a modular image analysis workflow environment in which individual analysis methods are considered as components of a pipeline and can be joined together seamlessly. Even though the role of data analysis workflow systems in genomics analysis has been recognized (Almeida, 2010), in the biological image analysis there are currently no image analysis focused integrated workflow systems available (Eliceiri et al., 2012).

We describe here an open source and modular analysis workflow system, Anima (ANduril IMage Analysis), for development of comprehensive image processing and multivariate statistical analysis of image data. Anima allows the combination of tools from different fields of informatics to a coherent workflow environment. It is targeted mainly for analysis and algorithm developers. Once an application is built with Anima, it may be executed by a wider audience. The main design principles are to enable rapid development and incorporation of new methods, without the need to port them from their original implementations. With Anima, an image analysis developer may systematically test different methods and include newly published ones regardless of the programing language the methods have been implemented in.

In order to gain scalability and flexibility, Anima has been designed to maximize portability, which rules out any server based frameworks (Rex et al., 2003; Berthold et al., 2007; Bauch et al., 2011; Rouilly et al., 2012). In addition, to remove the need of tedious porting to a specific language (HIPI, 2014), the framework needs to be able to run processes or scripts created with different languages. Thus, the most suitable framework architecture engine is the generic pipeline based solution (Ovaska et al., 2010; Wilde et al., 2011). Anduril is a generic workflow engine that contains a large library of bioinformatics related functionality and integration to several programing languages. Accordingly, Anima allows both rapid and flexible image data analysis as well as the use of powerful statistical methods. We demonstrate here the utility of Anima via two case studies. They display Anima in testing segmentation methods published on multiple, otherwise incompatible, platforms. In addition, we show seamless integration of image data extraction to a standard supervised prediction algorithm. Anima is an open source project available at http://www.anduril.org/anima.

## 2 Implementation

### 2.1 Specifications

The major design principles in creating Anima framework were that it integrates new algorithms with minimal implementation effort, it is command prompt friendly, and focuses on batch processing. To reuse existing software, the framework was built to extend an established pipeline engine, that:

1. provides a convenient mechanism to integrate existing software, not just libraries, as components of pipelines.
2. maintains the datatypes of the data flow, by preventing the wrong type of files being sent to a component.
3. creates an abstraction barrier between the programing code and the analysis design.
4. has a rich collection of existing data analysis components.
5. automatically parallelizes independent parts of the pipeline.
6. provides dynamic pipeline branching, to adjust the pipeline based on input data
7. does not rely on a server-client architecture, for easy deployment of smaller pipelines.
8. is able to resume analysis in the event of failures, prevents running of parts that have been already run, and reports and helps to solve failures.

Anima, in addition, extends the pipeline engine with:

1. the ability to use the image formats generated by majority of microscope image acquisition software.
2. interfaces for popular image analysis platforms, to further easen the integration.
3. convenience components for the commonly used parts of a typical image analysis.
4. file data types needed to represent the common image analysis objects.

The specifications are met by using the Anduril workflow engine (Ovaska et al., 2010) that facilitates the flow handling. Anduril engine executes components of an analysis pipeline and provides a simple workflow configuration language (AndurilScript). The components in the Anduril workflow are separate programs that in principle could be run independently and can be of any of the languages that have an application programing interface (API) in Anduril, such as Java, Bash, Perl, Python, R, or MATLAB, or in principle, any binary launched from the command line. The API includes a set of tools to read and write standard data types defined in Anduril. With the AndurilScript language, the user sets data sources and parameters and connects data to components, while Anduril engine passes the parameters on to the components and starts the processes. AndurilScript is a very simple language that abstracts the data flow and does not contribute to the low level processing itself. The analysis developer can concentrate on creating the data flow and see which tools are used, while the algorithm developer creates the components, which can be used in many different analyses.

Anduril engine makes development fast, since it stores the state of the analysis, allowing continuation where the processing ended in the case of a failure or other interruptions. The same mechanism also prevents any reruns of the parts of the analysis that do not require rerunning. This evaluation style is similar to lazy evaluation, like found in Haskell (2002), but forward propagated. In addition, it provides a syntax check for the component inputs, and file level datatyping. Any error situation is reported in a log file, and on the standard output.

### 2.2 Anima architecture

**Figure 1:**
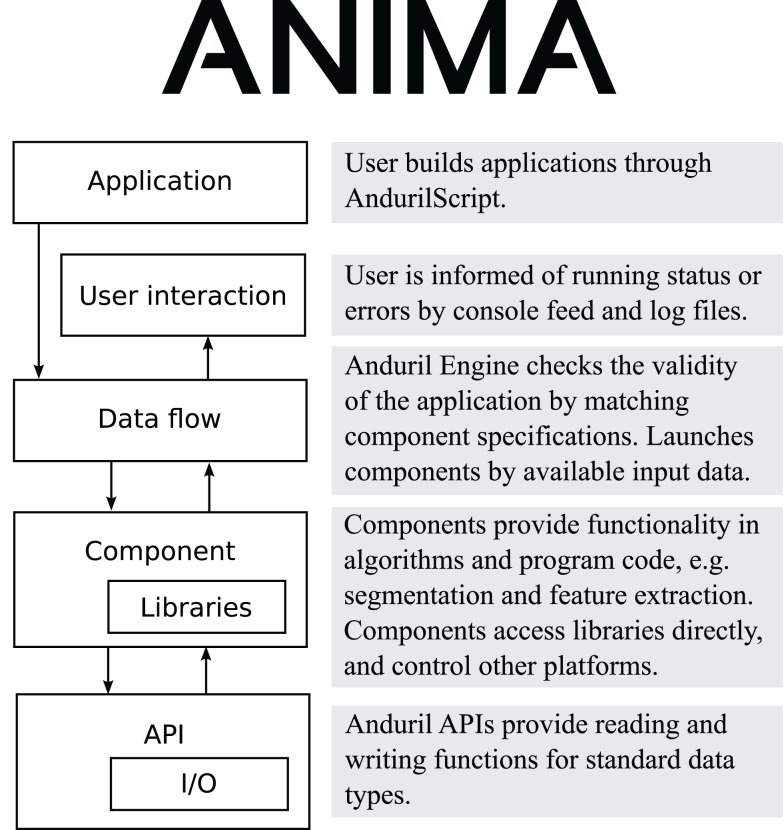
**Anima software architecture diagram**. Anima extends Anduril architecture by adding more functionality to multiple layers.

Anima is built on top of Anduril, and therefore shares its architecture. The architecture is visualized in Figure 1. At the top level of the architecture is AndurilScript, the language, the user uses to build applications. Anduril Engine is the core Java software that parses the script, checks the syntax, and creates the data flow network. The engine also inspects the component interfaces for standard data types, ensuring the data flow is sensible in terms of file types. The components and libraries provide the actual data processing algorithms. They are programs developed with different languages. Anduril provides the components with API functions that help the component developers in reading and writing the standard data types.

Anima and Anduril are designed with extreme programing philosophy (Beck and Andres, 2004). Both of them are continuously developed with small steps, and have collective code ownership. The engine and components are required to have test cases, the design encourages test driven development, and the test cases are constantly run in a continuous build environment. Most of the developers are also heavy users of the system, creating a very close developer-customer relationship.

Anima extends Anduril by adding more functionality to the component and API levels of the architecture. The APIs are extended by image data handling functions. The most significant extension to Anduril is the selection of components that provide either a convenient functionality, like segmenting images or extracting features, or give access to a freely scripted platform, such as ImageMagick (2014) or Fiji. Currently, we provide 48 components, which are updated as features are required or requested. The latest versions are always available in the public code repository. The components are fully documented and accessible in a searchable component reference at http://anduril.org/pub/bundles/image_analysis/doc/.

### 2.3 Data flow in Anima

**Figure 2:**
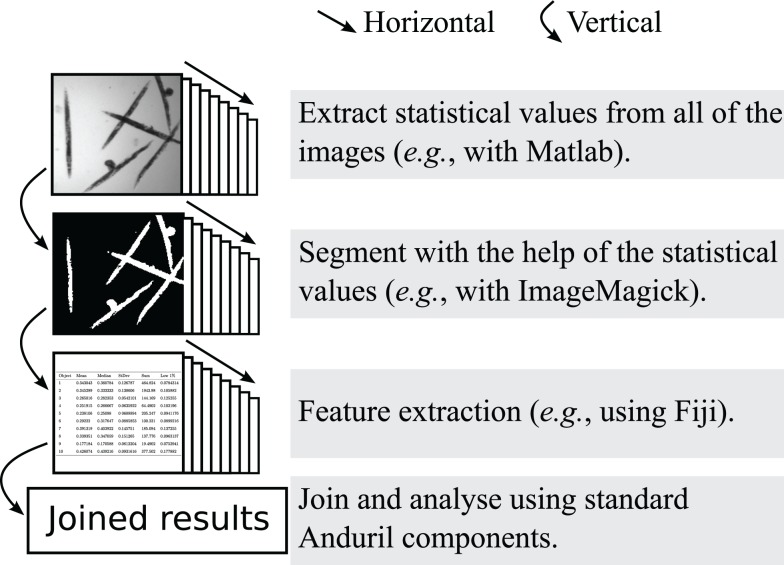
**An example of the horizontal data flow, where images are segmented and extracted for numerical features**. The horizontal data flow allows the inspection of the suitability of the thresholding step for all of the images, before continuing with the time consuming feature extraction.

The main design concept of Anima is to function as a super platform, i.e., to take advantage of existing software and not to replace them. Anima is ideal for developing and testing new methods, while it can also be used to run established standard analysis. Anima encourages horizontal data flow schematic, which means that each step (e.g., segmentation) is processed with all of the images, as shown in Figure 2. Horizontal data flow ensures that each step produces sensible results before performing the downstream analyses, for example setting segmentation parameters that are suitable for all of the images. Anima is designed to comply with the concepts of openness and usefulness as suggested by Carpenter et al. (2012).

Processing images is often case specific, and thus Anima provides components that allow the user to execute custom procedures in popular image processing tools. For example, several standard procedures for processing microscopy images, such as background subtraction and overlaying gray scale images to color images, have been added. The more complex steps (e.g., segmentation and feature extraction) have been written as components that contain several individual methods and their parameters. The components and their parameters are documented in an easy-to-search HTML page structure. Highlights of components and application pipelines included in Anima that are directly useful in image analysis are given in Table 1.

**Table 1:** **Component categories and their applications in the Anima bundle**.

Task	Description
Segmentation	A set of segmentation tools provided cover majority of cases. Masks can be created by global, local, or seeded algorithms. Masks can be further fine tuned through shape filtering or active contour algorithm (Kumar, 2010). Ellipses and line-objects are created by separate components.
Object management	Objects in an image mask can be removed by any measured or calculated feature value. For example, by clustering the values of intensity and area, the objects that belong to a cluster where both values are small can be removed from the mask images.
Object relation	Any two masks can be related to each other. For example, a cell contains one or more nuclei. The cell and its nuclei have a parent–child relation. Further, nucleoli can be related to parent nucleus, creating a chain of primary, secondary, and tertiary objects.
Feature extraction	Anima has a pre-defined set of intensity, texture, and morphological features that can be extracted with different components. Features can be extracted from masks, ellipses, and line object representations.
Visualization	These components can add annotations on images from tabular data or merge images and color mask objects by clusters or other values. Time lapses or time varying plots can be rendered in GIF animations or common video formats. A web site publisher exists to present the result data and visualizations.

The executed components are separate processes launched from the Anduril engine. Thus, the steps that are independent can be launched simultaneously and in parallel. Accordingly, parallelizing processes allows to fully utilize the modern processors’ multicore and multithreading technologies or to send the processes to a cluster computing environment. Two cluster environments, SLURM (Yoo et al., 2003) and Oracle Grid Engine (Oracle Inc., CA, USA), have been successfully tested with Anima.

### 2.4 Software integration

Anima has the advantage of supporting multiple programing languages. It is straightforward to take an existing software or library and merge it to the workflow. In addition to the standard Anduril API collection, Anima provides environments to run CellProfiler workflows, as well as MATLAB, Fiji, and ImageMagick scripts. Anima is not dependent on any of the other platforms – only the ones the developer chooses to use.

A key issue in using multiple software concurrently is the definition of file formats in component input/output relations. The communication between components in an Anima workflow is done via files. Typically, the file is a table of values in the Comma Separated Values (CSV) format or an image file in the Portable Network Graphics (PNG) format. Anima can process various common image formats, but PNG is preferred because it is a flexible and open source image format. It supports many bit depths and provides lossless compression, which is especially suitable for two color mask images. Anima provides a converter from all major image formats via the Bio-Formats library (Linkert et al., 2010). Since the image processing steps are typically fast (a few seconds per image), the components mediate results by folders of images, not as individual image files. This approach reduces the overhead of starting new processes and is the basis of horizontal data flow.

To enable complex analyses, Anima introduces two standard object representations in addition to conventional black and white mask images. The first one is the MaskList, which is a folder of images. Each image represents a single object mask, allowing non-connected and overlapping objects. The second one is an Ellipse CSV or a table of ellipse coordinates. The ellipse is a compact and robust model for various shapes, such as cell nuclei, in molecular biology. The ellipses are used in the description of Gaussian Blob objects as explained in the feature detection schema presented by Lindeberg (1998).

In our case study, we show integration of the WEKA [Waikato Environment for Knowledge Analysis (Hall et al., 2009)] software to the image analysis pipeline. WEKA provides access to a number of machine learning implementations in Java, which are included in the Anduril default installation. We use here a Naïve Bayesian Classifier, a classic machine learning algorithm (John and Langley, 1995), and the Random Forest algorithm, which is a powerful ensemble classification algorithm (Breiman, 2001).

### 2.5 Scripting

Anima takes advantage of the Anduril workflow engine, which is based on command line interface. It allows the design and implementation of scalable and flexible analysis workflows. Even though most of the image processing and analysis tools are graphical user interface based, they can typically be scripted and run from the command line. Anima is controlled with the AndurilScript language. An example script extracting morphological features from labeled cell nuclei is presented in Table 2.

**Table 2:** **An example pipeline script extracting morphological features by segmenting cell nuclei**.

/* Define a folder of RGB color images. */
list = INPUT(path = “path/to/images”)
/* Extract the blue channel. (3rd) */
blue = ImageExtract(dir = list, ch = 3)
/* Cell segmentation based on the blue channel,
finding round shapes with areas of 200–2000
pixels. */
cells = ImageSegment(dir = blue.channel,
method = “Shape”,
minround = 0.90, maxround = 1,
minsize = 200, maxsize = 2000)
/* Extract morphological features. */
morph = ImageMorphFeatures(mask = cells.mask)
/* Create a visualization to confirm the segmentation.
* Blue signal with 460nm wavelength color.
* Object mask perimeter lines with white color. */
visualization = ImageRGBMerge(dir1 = blue.channel,
color1 = 460,
dir2 = cells.perimeter,
color2 = “W”)
/* Create a brief summary, averaging the features
of each image. */
summary = CSVSummary(csv = morph.table,
summaryType = “mean”,
clusterCol = “File”)

AndurilScript allows the data analyst to have a permanent record of all steps used in the analysis. This, together with saving the original images, is crucially important in image processing analysis (Nature, 2006, 2013; JCB, 2013). Furthermore, as parts of a script can be easily transferred to a new project, the scripting approach offers an easy solution to efficient code reuse. Since the scripts are conventional text files, and the intermediate results standard CSV and image files, the developer may use any of their favorite tools to work with Anima.

It should be emphasized that AndurilScript is not a new programing language replacing others in the analysis. The image processing is executed in components running the original code in their respective languages. AndurilScript is only used to control the flow of data between the components.

An important feature in Anduril, not generally found in workflow systems, is the ability to use for-loops, which allows several options to make advanced pipelines. For example, scripting with for-loops can be used to test a method over a range of parameter values, test a range of different implementations for the same method, or parallelize a computationally heavy analysis.

## 3 Materials and Methods

### 3.1 Data description for case studies

We provide two case studies of the use of Anima with the benchmark data available at the Broad Bioimage Benchmark Collection (Ljosa et al., 2012).

In the first case study, the source image dataset (BBBC005v1) contains 9,600 synthetic cell images (600 images × 16 different simulated levels of out-of-focus effect). This dataset is ideal for testing segmentation methods’ robustness for incorrect focus in a high-throughput application.

For the second use case, we used the *C. elegans* infection live/dead image set BBBC010v1. The set consists of 97 bright field microscopy images annotated to belong to either live or dead class. The annotation makes the dataset ideal for testing machine learning applications.

### 3.2 Derived measures in the case studies

In the first case study, the aim is to compute the number of correctly segmented objects. Segmentation methods, watershed in particular, may oversegment the objects producing more objects than the actual count. Thus, direct comparison of count (*measured count*/*validation count*) may lead to *ratio* >100%, which hinders interpretation of the results. Therefore, we used the non-incorrect object ratio that overcomes this issue:

(1) $$1-\frac{\|measured\;count\;–\;validation\;count\|}{validation\;count}.$$

**Figure 3:**
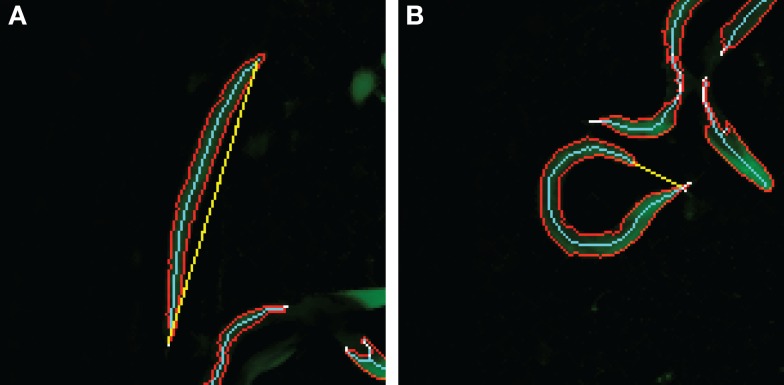
**Distance-length ratio in a straight/dead (A) and curved/alive (B) *C*. elegans**. The values for the DLR are **(A)** 116/112 = 1.04 and **(B)** 21/104 = 0.20. The skeleton length is represented by the number of pixels in the skeleton, and therefore, the ratio may exceed 1. The cyan line represents the skeleton length, and the yellow line the end-to-end length.

In the second case study, the objective is to classify images based on the curvature of worm-like objects. This requires two measurements: the length of an object measured along its center line and the distance between the end points of the object as shown in Figure 3. With these two values, we can construct a derived feature; the distance-length ratio (DLR) that describes normalized curvature,

(2) $$DLR=\frac{end\;–\;to\;–\;end\;distance}{skeleton\;length}.$$

## 4 Results

We show two case studies developed with the Anima workflow system. In the first case, the objective is to segment objects in a large number of images using several existing algorithms. This example demonstrates the use and parallelization of existing platforms Fiji, MATLAB (Mathworks Inc., MA, USA), and FARSIGHT (Bjornsson et al., 2008), without the need of re-implementation of the algorithms. In the second case, the workflow objective is to predict whether *C. elegans* worms are dead or alive based on features extracted from images. This case displays the integrated use of image processing with MATLAB, data processing with CRAN R (Ihaka and Gentleman, 1996) and supervised machine learning with WEKA library (Hall et al., 2009). All data and Anima scripts are available in Supplementary Material.

### 4.1 Case study I: High-throughput segmentation

**Figure 4:**
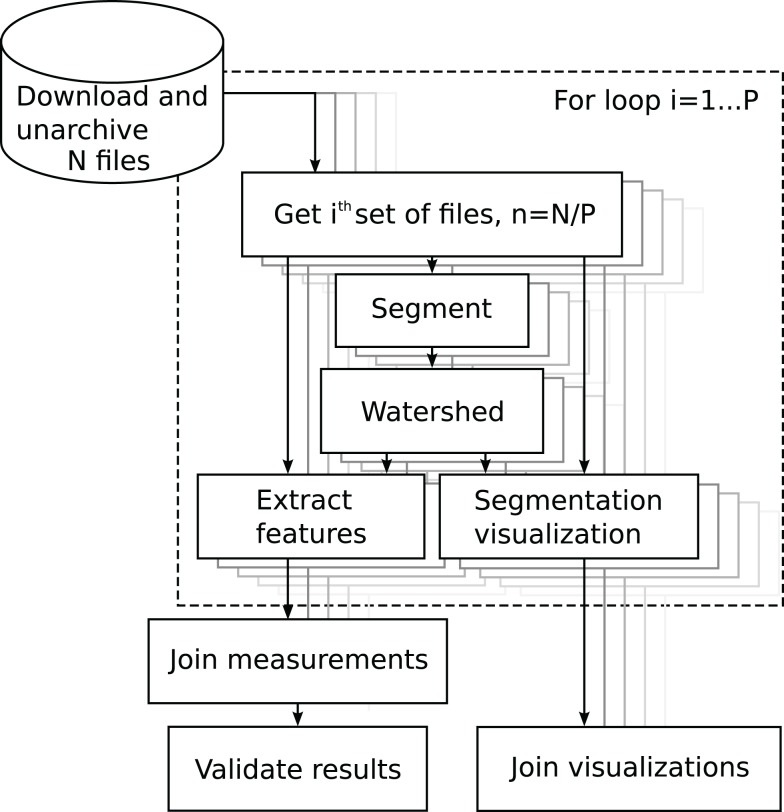
**Block diagram of the analysis of the BBBC005 image set**. The source folder contains *N* files. The user sets the partitioning constant *P* to, e.g., the number of processor cores. The for-loop, in dashed rectangle, iterates over index *i*, automatically parallelizing the components.

Segmentation is one of the most crucial operations in biomedical image analysis. It establishes the measurement of the objects of interest. Here, we conduct cell nucleus segmentation and counting using three different image analysis platforms. The pipeline segmented, counted, and produced visualizations of the segmentations by overlaying the mask perimeter on the original signal image. The process diagram is shown in Figure 4.

The first of the three platforms used here was Fiji. The nuclei were segmented with the Global Otsu thresholding method (Otsu, 1979), corrected with a constant multiplier of 1.3, which was set by visual inspection. The thresholding was followed by a watershed. We compared the results of the Otsu segmentation to a graph cut method developed by Al-Kofahi et al. (2010) and a wavelet based segmentation developed by Padfield et al. (2011). The graph cut method is implemented in C and distributed as an executable binary with the FARSIGHT toolkit, whereas the wavelet method is distributed as a MATLAB function.

**Figure 5:**
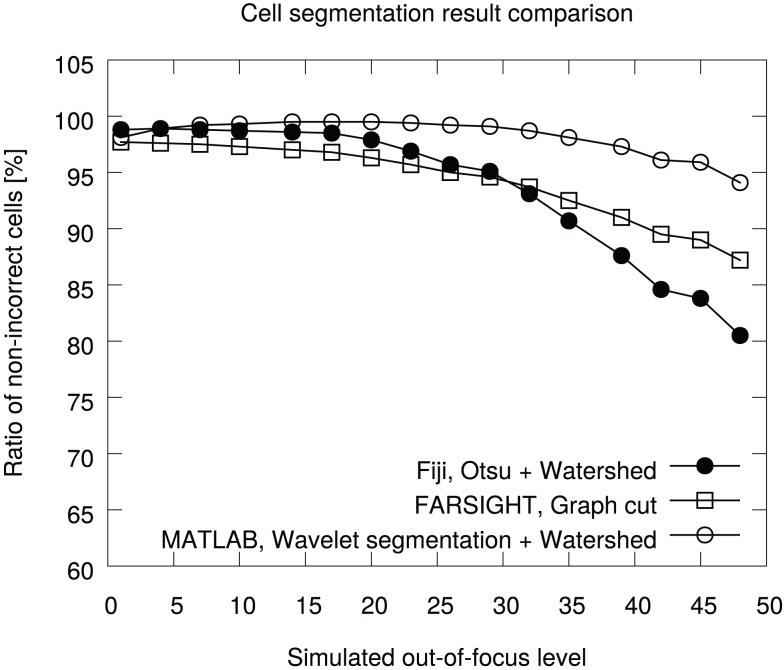
**Comparison of segmentation accuracies in the BBBC005v1 benchmark set**. The ground truth contains 30,300 cells for each focus level. The graph is generated in the pipeline analyzing the data.

The ratios of non-incorrect cells (Equation 1) with each focus parameter are shown in Figure 5. On average, the Fiji Otsu segmentation accuracy was 94% of the number of objects in the annotation. For comparison, the graph cut approach accuracy was 94% and the wavelet method accuracy 98%. The out-of-focus affects Otsu segmentation the most and the accuracy starts to drop quickly. In contrast, the Wavelet method is very robust to misfocus.

Segmentation of 9,600 images with the three methods used in this case study took 5 h 6 min wall-clock time using a single thread process on a 3.40 GHz clock speed Intel i7-2600 processor. The benefit of using Anima, instead of running the tasks with Fiji directly, is that Anima provides tools to partition the data and to use parallelized computation. When using hyper-threaded four core CPUs with six threads and partitioning the data in 24 parts to maximize parallelization, the analysis lasted 1 h 44 min. In addition, when running the analysis on a three node SLURM cluster, where each node runs 24 threads on 24 cores, the running time decreased to 1 h 2 min. The running time does not decrease further with increased parallelization, due to increase of communication through the filesystem. The full pipeline, providing the images and validation statistics, is available in Supplementary Material.

The inherent property of Anima using Anduril is to speed up development via running only the parts of analysis that are affected after a change in the pipeline configuration. For example, if the developer wants to use a different metric for the validation statistics, re-executing the pipeline after modification takes mere seconds to run, as only the very last few steps would be processed.

### 4.2 Case study II: Prediction of *C. elegans* vital status

Predicting whether a *C. elegans* worm is dead or alive from images requires automated image processing and the use of machine learning methods. The phenotype description suggests us that live worms appear curved, while the dead ones are mostly straight (Figure 3). Thus, we first segmented and skeletonized the brightfield images and then measured the skeleton features.

To describe the morphologies of the skeleton features in an image, we calculated two image descriptors: the median of end-to-end distances and distance-length ratios. The two-dimensional value was then used as the training value for a Random Forest classifier. Half of the images (48 images) were used in training and the other half in validation.

Out of the 48 validation images, only one was predicted wrong with the Random Forest classifier, leading to ROC area under curve (AUC) of 0.979. In comparison, the Naïve Bayesian classifier produces the same AUC of 0.979.

The pipeline uses MATLAB, CRAN R, and WEKA based components provided by Anima and Anduril. The running time on an Intel i7-2600 processor, using two threads, was 3 min. The full pipeline analyzing the images, training, and validating the classifiers with their parameters, is available in Supplementary Material.

## 5 Discussion and Conclusion

We have introduced here Anima, which is a modular image analysis focused workflow system. Instead of being a monolith software that consists of complex complete solutions for image analysis applications, Anima is a flexible, scalable, and extendable modular platform. The main benefit of developing with a modular platform design is the easiness of adding any new functionality the developers find or develop themselves, independent of the platform the method is implemented on. Anima is designed to maximize reusability. The existing components for well-established procedures, such as segmentation, come with easily modifiable source scripts. To this date, Anima has been used in several image analysis applications as shown Table 3.

**Table 3:** **Applications built with Anima**.

Application	Description
Computer aided analysis	Extracting features from a manually segmented set (Cheng et al., 2011; Jalkanen et al., 2012)
Granular objects and neighbors	Exploring novel analysis methods by using granular point-like objects and their neighbors for data source (Blom et al., 2012).
Alternative segmentation	Finding Gaussian Blob-like objects (Aarne et al., 2013)
Time-series	Time lapse cell analysis with time-series data analysis (Moore et al., 2011)
Decision making	Using machine learning for a subjective decision maker (Enzerink et al., 2010; Jäämaa et al., 2010, 2012)
Artificial image source	Protein array image segmentation, quality metrics and analysis (Savilahti et al., 2010)

The use of command prompt and scripting requires some computer science background. Thus, Anima is targeted mainly for algorithm and analysis developers. Anima is best suited for rapid and systematic development of novel methods, combining tools from existing platforms, prototyping a full analysis pipeline, and systematic testing of analysis methods. While composing an analysis pipeline requires some computer science expertise, the use and modification of existing pipelines, and their parameters is easy even for non-experts.

The flow architecture in Anima is horizontal, which has several advantages over the more common vertical flow, in which each image is processed through the whole pipeline before moving on to the next image. Horizontal flow allows us to make sure the processing in each step produces sensible results before performing downstream analyses. Furthermore, it allows the measurement of features based on all images and the use of them later on in the analysis workflow. For instance, it is possible to choose a threshold level based on the mean of intensities throughout all of the objects detected in the workflow.

When any framework is wrapped around the actual programs needed to process data, overhead is presented. With Anduril, the overhead in the computer memory is large compared to conventional scripting environments, such as Python or Perl. The memory overhead is mainly due to the loading of Java Virtual Machine and building the component repository; a list of all the available components with their data types and parameters. When starting a run, the data flow network is built and checked for file and parameter data type consistencies and the current state is saved. These steps require time, although typically only a few seconds. Because of the consistency check, Anduril prevents illegal calls of components. On the other hand, one of the major benefits of implementing Anima on Anduril, compared to many other scripting environments, is that Anduril runs only the parts that need running. The decision to run may be because of changed input files, changed parameters, or an error returned by a component in a previous execution. The overhead comparison of Anima versus Bash scripting in the Supplementary Material demonstrates the time saved by using Anima.

The communication between components in Anduril is done via files. While the file-based communication between the components may be slower and require more hard disk space than keeping everything in memory, the benefit is gained during the development of the workflow – any change in the configuration apply only to changed components, which allows the skipping of steps that have already been processed. Since each step is saved, it is easy to inspect their intermediate results and change parameters accordingly. The file-based communication allows the Anima to be a superplatform because different scripting or programing languages can be used simultaneously. The drawback of file-based communication is that in a cluster environment Anduril has to rely on a shared network file system approach.

The ability to run the most popular platforms within one environment ensures that the developers do not have to spend time porting existing methods to the language of their working environment. Even if an interesting novel method is not implemented in these platforms, it may be used as an executable binary, which again is easy to incorporate into Anima. In addition to being a super platform that allows executing third-party software, several common image processing tasks can be completed with the existing Anima components.

## Conflict of Interest Statement

The authors declare that the research was conducted in the absence of any commercial or financial relationships that could be construed as a potential conflict of interest.

## Supplementary Material

The Supplementary Material for this article can be found online at http://www.frontiersin.org/Journal/10.3389/fbioe.2014.00025/abstract
