# Supplementary material for: Anima: Modular Workflow System for Comprehensive Image Data Analysis
Source: Front Bioeng Biotechnol. 2014 Jul 30;2:25. doi: 10.3389/fbioe.2014.00025 (PMC4115631; doi:10.3389/fbioe.2014.00025)
Supplement: Supplementary file 1 [file Data_Sheet1.PDF]

Frontiers in Bioengineering

Methods
10 July 2014

Supplementary Material: Anima: Modular workflow system for comprehensive image data analysis

Ville Rantanen [1], Miko Valori [1] and Sampsa Hautaniemi [1,*]

[1] *Research Programs Unit, Genome-Scale Biology & Institute of Biomedicine, Biochemistry and Developmental Biology, University of Helsinki, Finland*

Correspondence*:
Sampsa Hautaniemi
Research Programs Unit, Genome-Scale Biology & Institute of Biomedicine, Biochemistry and Developmental Biology, University of Helsinki, Finland,
sampsa.hautaniemi@helsinki.fi

1 SUPPLEMENTARY DATA

Here, we present full pipelines for processing two use cases with the Anima workflow system.

Anima is a modular workflow environment for comprehensive analysis of image data. Anima uses the Anduril workflow engine to facilitate the flow handling. Anduril engine executes components of an analysis pipeline and it provides a workflow configuration language (AndurilScript). The components in the Anima workflow are separate programs that in principle could be run independently and can be of any of the languages that have an application programming interface (API) in Anduril, such as Java, Bash, Perl, Python, R, or MATLAB. With the AndurilScript language the user sets data sources and parameters, and the way different components are connected, while Anduril engine passes the parameters on to the components and starts the processes.

2 CASE STUDY I: HIGH-THROUGHPUT SEGMENTATION

Here, we conduct cell nucleus segmentation and counting using Fiji through Anima. The nucleus segmentation was done with the Global Otsu thresholding method, corrected with a constant multiplier of 1.3, which was set by visual inspection. The thresholding was followed by a watershed. Further, the pipeline produced visualizations of the segmentation by overlaying the mask perimeter on the original signal image.

We compare the results to the segmentation with a graph cut method developed by Al-Kofahi and a wavelet based segmentation developed by Padfield. The graph cut method is implemented in C and distributed as a compiled binary with the FARSIGHT toolkit, where as the wavelet method is distributed as a MATLAB function.

Segmentation of 9,600 images with the three methods used in this case study took 5 hours 6 minutes wall-clock time using a single thread process on a 3.40GHz clock speed Intel i7-2600 processor. The benefit of using Anima, instead of running the tasks with Fiji directly, is that Anima provides tools to partition the data and to use parallelized computation. When using hyper-threaded four core CPUs with six threads and partitioning the data in 24 parts to maximize parallelization, the analysis lasted 1 hour 44

minutes, respectively. In addition, when running the analysis on a three node SLURM cluster, where each node runs 24 threads, the running time decreased to 62 minutes.

The running times do not include the time required to download data.

Listing 1. High-throughput segmentation pipeline

```
/* This first part defines variables, functions and scripts to be used
 * later in the script
*/
// Download the data set
filedownload=URLInput(url="http://www.broadinstitute.org/bbbc/BBBC005/BBBC005_v1_images.zip",
    @execute="once")
// Custom script to extract the archive
fileunarchive=BashEvaluate(var1=filedownload, script="cd @folder1@; unzip -j @var1@; mv *w1.TIF
    @folder2@")

// Define image input
filedir=fileunarchive.folder2

// Defines how many parts the data is split in to
parals=24

// Fiji script to watershed a mask
//   Returns the mask in output port 'dir1'
//   and the perimeter of the mask in port 'dir2'
watershed_script='''
IJ.run(im1, "8-bit", "");
IJ.setThreshold(im1,127, 255)
IJ.run(im1, "Convert to Mask", "")
IJ.run(im1, "Fill Holes", "")
IJ.run(im1, "Watershed", "")
imout=im1
imout2=imageCopy(im1)
IJ.run(imout2, "Outline", "")'''

// This function calculates a difference between the number of
// segmented objects and actual number
function validation(CSV meas,boolean summary=false)->(CSV sum) {
    if (!summary) {
        measurements_summary=CSVSummary(csv=meas,clusterCol="File")
    } else {
        measurements_summary=meas
    }
    measurement_validation=CSVTransformer(csv1=measurements_summary,
        transform1="as.integer(gsub('SIMCEPImages_.*_C([0-9]+)_F.*','\\\\1',csv1[,'File']))",
        transform2="csv1[,c('Count')]",
        transform3="as.integer(abs(transformed[,1] - csv1[,'Count']))",
        columnNames="c('RealCells','Count','Error')")
    validation_summary=CSVSummary(csv=measurement_validation, summaryType="sum", counts=false)
    // Here is the calculation of non-incorrect ratio
    validation_ratio=CSVTransformer(csv1=validation_summary, transform1="csv1",
                      transform2="round(1000*(1-(csv1[,3]/csv1[,1])))/10",
                      columnNames="c(colnames(csv1),'Correct ratio')")
    return record(sum=validation_ratio)
}
/* In this part, we run the analysis, and parallelize with for-loops
*/

visuals={}
numeric={'FJ'={}, 'FS'={}, 'WL'={}}

for x:std.range(1,parals) {
    // fetch [parals]:th part of the data set (gray scale images)
```

```
    file_split=FolderSplitter(dir=filedir,N=parals,subset=x,
                              link=false,order="file",@name="file_splitX"+x,@keep=false)
    // Segment the images with Otsu algorithm, and correct the level by a constant.
    // The correction is set by visual evaluation by running only one
    // parallel thread first.
    FJnuclmask=ImageFijiSegment(force dir=file_split.dir1,
                         method="Otsu dark",
                         corr=1.3,
                         @name="FJnuclmaskX"+x)
    // Watershed the mask
    FJwaterprocess=ImageFijiOperation(
                         dir1=FJnuclmask.mask,
                         script=watershed_script,
                         @name="FJwaterprocess"+x)
    // Measure the features for each object
    FJfeatures=ImageFijiFeatures(mask=FJwaterprocess.dir,
                         force image=file_split.dir1,
                         @name="FJfeaturesX"+x)

    // Get masks with FarSight library
    FSnuclmask=ImageSegmentGraphCut(force dir=file_split.dir1,
                      max_scale=12,
                      min_scale=10,
                      @name="FSnuclmaskX"+x)
    // Make sure mask is valid 8-connected for Fiji
    FSnuclsep=ImageMagickOperation(dir1=FSnuclmask.mask,
                      command="@in1@ -morphology Erode Disk:1 @out@",
                      @name="FSnuclsepX"+x)

    // Measure the features for each object
    FSfeatures=ImageFijiFeatures(mask=FSnuclsep,
                         force image=file_split.dir1,
                         @name="FSfeaturesX"+x)
    // Get masks with Wavelet segmentation library (requires MATLAB)
    WLnuclmask=ImageSegment(force dir=file_split.dir1,
                      method="Wavelet", clearborders=false, fillholes=false,
                      waveletparam="4,4",
                      @name="WLnuclmaskX"+x)
    // Watershed the mask
    WLwaterprocess=ImageFijiOperation(
                         dir1=WLnuclmask.mask,
                         script=watershed_script,
                         @name="WLwaterprocess"+x)

    WLfeatures=ImageFijiFeatures(mask=WLwaterprocess.dir,
                         force image=file_split.dir1,
                         @name="WLfeaturesX"+x)
    // Create an RGB visualization of the grayscale image, and the
    // segmentation perimeter. Change to PNG for smaller size.
    // For brevity, thesea are done with ImageMagick, instead of Fiji
    compose_command="-compose Plus @in1@ \\( @in2@ -color-matrix ' 0 0 0 1 1 1 0 0 0 ' \\) -composite
        @out@"
    FJvisu=ImageMagickOperation(force dir1=file_split.dir1, dir2=FJwaterprocess.dir2,
                     oldExtension=".TIF", extension=".png",
                     command=compose_command, @name="FJvisuX"+x)
    FSvisu=ImageMagickOperation(force dir1=file_split.dir1, dir2=FSnuclmask.perimeter,
                     oldExtension=".TIF", extension=".png",
                     command=compose_command, @name="FSvisuX"+x)
    WLvisu=ImageMagickOperation(force dir1=file_split.dir1, dir2=WLwaterprocess.dir2,
                     oldExtension=".TIF", extension=".png",
                     command=compose_command, @name="WLvisuX"+x)
    visu=ImageMagickOperation(dirArray={FJvisu,FSvisu,WLvisu}, binary="montage",
                     command="@inall@ -tile x1 -geometry +3+0 @out@", @name="visuX"+x)
```

```

    // Remove objects where pixel area is less than 200
    FJfeatures_filt=CSVFilter(csv=FJfeatures.table, lowBound="Area=200",
                         @name="FJfeatures_filtX"+x)
    FSfeatures_filt=CSVFilter(csv=FSfeatures.table, lowBound="Area=200",
                         @name="FSfeatures_filtX"+x)
    WLfeatures_filt=CSVFilter(csv=WLfeatures.table, lowBound="Area=200",
                         @name="WLfeatures_filtX"+x)
    // Store results for later joining.
    visuals[x]=visu.dir
    numeric['FJ'][x]=FJfeatures_filt
    numeric['FS'][x]=FSfeatures_filt
    numeric['WL'][x]=WLfeatures_filt
}

measurements={}
measurements_grouped={}
measurements_split={}
validations={}
method={}
method_sorted={}
method_rename={}
for s:std.split("FJ,FS,WL",",") {
    // Join the measurement CSVs from the parallel sets
    measurements[s]=CSVListJoin(files=numeric[s], fileCol="")
    // Parse the filename to find what is the simulated off-focus level
    // This is the final result from the analysis
    measurements_grouped[s]=CSVTransformer(csv1=measurements[s],
        transform1="csv1",
        transform2="as.integer(gsub('SIMCEPImages_.*_F([0-9]+)_s.*','\\\\1',csv1[,'File']))",
        columnNames="c(colnames(csv1),'Focus')")
    // Split the results by off-focus level
    measurements_split[s]=CSVSplit(csv=measurements_grouped[s], labelCol="Focus")
    // Validate each of the focus levels separately
    validations[s]={}
    for el:std.iterArray(measurements_split[s].array) {
        validations[s][el.key]=validation(meas=measurements_split[s].array[el.key])
    }
    // Join validations in one table
    method[s]=CSVListJoin(files=validations[s], fileCol="Focus")
    // Sort CSV by Focus level
    method_sorted[s]=CSVSort(csv=method[s])
    method_rename[s]=CSVFilter(csv=method_sorted[s],
                          rename="Correct ratio="+s)
}

// Join results
methods_compare=CSVJoin(array=method_rename,
                        intersection=false,
                        keyColumnNames="Focus")

// Plot a graph with non-incorrect ratios.
// GNUPlot can be found in the Tools bundle: https://code.google.com/p/anduril.tools/
focus_plot=GNUPlot(csv={methods_compare},
            script='''
    set terminal pdf font 'Helvetica,11' size 8cm,7cm
    set output @output_segmentation_compare.pdf@
    set title "Cell segmentation result comparison"
    set ylabel "Ratio of non-incorrect cells [%]"
    set xlabel "Simulated out-of-focus level"
    set key right bottom
    set yrange [60:105]
    set style line 1 lc rgb '#000000' lt 1 lw 2 pt 7 ps 0.7
```

```
    plot @input_1@ using 1:5 title "Fiji , Otsu + Watershed" with lp ls 1 pt 7,\
         @input_1@ using 1:6 title "FARSIGHT, Graph cut" with lp ls 1 pt 4,\
         @input_1@ using 1:7 title "MATLAB, Wavelet segmentation + Watershed" with lp ls 1 pt 6''')

// Join the visualization images from the parallel sets
visualization=Array2Folder(array=visuals , fileMode=".", link=false)
```

3 CASE STUDY II: PREDICTION OF *C. ELEGANS* VITAL STATUS

Predicting whether a *C. elegans* worm is dead or alive from images requires automated image processing and the use of machine learning method. The phenotype description tells that live worms appear curved, while the dead ones are mostly straight. Thus, the pipeline first segments and skeletonizes the brightfield images and then measures the skeleton features.

The pipeline uses the morphologies of the skeleton features in an image to create an image based classifier. The values are then used as the training set for a random forest classifier. Half of the images (48 images) are used in training and the other half in validation.

Listing 2. *C. elegans* curvature assay pipeline

```
// Download the data set
filedownload=URLInput(url="http://www.broadinstitute.org/bbbc/BBBC010/BBBC010_v1_images.zip")

// Custom script to extract the archive
fileunarchive=BashEvaluate(var1=filedownload, script="cd @folder1@; unzip -j @var1@")

//Define the folder of input images
filedir=fileunarchive.folder1
// create a list of files matching a pattern
transmitted=Folder2Array(folder1=filedir ,
                         filePattern="(.*w2.*tif)")

//------------------------------------------------------------
// Convert the images to grayscale PNG
signal=ImageExtract(array=transmitted ,ch=1,namestring="@ONAME@.png")
// Preprocess images, inverting color,equalizing intensity and background removal.
sigprocess=ImageMatlabOperation(force dir1=signal.channel ,
         script="foo=imclose(adapthisteq(mat2gray(imcomplement(im1))),strel('disk',2,0));"+
               "imout=(foo-medfilt2(foo,[21 21]));")
// Segment images with Otsu method, and constant correction, removing small clutter
wormask=ImageSegment(force dir=sigprocess.dir ,
                     method="otsu",minsize=200,
                     corr=0.6,fillholes=false ,
                     maxsize=0,minintensity=0.001)
// Thin the mask to skeletons
skeleton=ImageMatlabOperation(dir1=wormask.mask,
         script="imout=bwmorph(imclose(im1,strel('disk',2,0)),'thin',Inf);")
// Measure the features from skeletons
skfeatures=ImageSkeletonFeatures(skeleton=skeleton.dir ,
                    gray=sigprocess.dir ,
                    maxdist=150)
// Create a visualization of the segmentation and skeletonization
seg_visu=ImageRGBMerge(force dir1=sigprocess.dir ,color1="G",
                     dir2=wormask.perimeter ,color2="R",
                     dir4=skeleton.dir ,color4="C")
// Remove short skeletons from the data
measurements_filt=CSVFilter(csv=skfeatures.table , lowBound="Length=20")
// Calculate an additional feature from the measured
measurements_calc=CSVTransformer(csv1=measurements_filt ,
       transform1="csv1[,c('File','Distance')]",
```

```
        transform2="csv1[,'Distance']/csv1[,'Length']",
        columnNames="c('File','D','DLRatio')")
// Calculate per-image summaries
measurements_median=CSVSummary(csv=measurements_calc,
                    summaryType="median", clusterCol="File", counts=false)
// Parse the filename for ground truth
measurements_grouped=CSVTransformer(csv1=measurements_median,
        transform1="paste('G',as.integer(gsub('(.*)(..)(_w2).*','\\\\2',csv1[,'File'])>12),sep='')",
        transform2="csv1",
        columnNames="c('Group',colnames(csv1))")
// Split the data to training and validation sets
images_split=CSVSplit(csv=measurements_grouped, labelCol="File")

sets_split=ArraySplitter(array=images_split, N=2)
sets_visu=FolderSplitter(dir=seg_visu, N=2)

training_set=CSVListJoin(files=sets_split.array1, fileCol="")
validation_set=CSVListJoin(files=sets_split.array2, fileCol="")
// Train and classify with random forest classifier
rf_classifier=WekaClassifier(data=training_set,
               testdata=validation_set,
               classifydata=validation_set,
               classColumn="Group",
               columnsToRemove="File",
               crossValidation=500,
               methodClass="weka.classifiers.trees.RandomForest")
// Train and classify with naive bayes classifier
nbc_classifier=WekaClassifier(data=training_set,
               testdata=validation_set,
               classifydata=validation_set,
               classColumn="Group",
               columnsToRemove="File",
               crossValidation=500,
               methodClass="weka.classifiers.bayes.NaiveBayes")
// Create annotations for the classified images
annotations=CSVTransformer(csv1=validation_set,
                           csv2=rf_classifier.predictedClasses,
            transform1="csv1[,'File']",
            transform2="paste('Class:',csv2[,'Group'],'Prediction:',"+
            "csv2[,'PredictedClass'],'<br>',csv1[,'File'])",
            columnNames="c('File','Annotation')")
// Create a result HTML image gallery with annotations
gallery=ImageGallery(folderRoot=sets_visu.dir2,
                     csvRoot=annotations,
                     annotationCol="Annotation")
```

Table 1. Overhead resources comparison: Bash vs. Anima.

Script	Wallclock time [s]	CPU time [s]	Max memory [Mb]
Bash run 1	41	9	48
Bash run 2	43	10	48
Anima run 1	50	10	500
Anima run 2	15	5	500

4 OVERHEAD COMPARISON

Adding a framework around any process creates overhead in terms of processing time, and computer memory. If the framework can provide a logic that prevents the execution of unneeded processes, the overhead is reduced. Anima on Anduril provides such a logic.

Assume a user wants to first blur and then resize a set of images. The user creates a script to do so, but after running it, realizes the resize scale must be changed. In any conventional scripting language the blurring phase is run again, whereas in Anima only the part of the pipeline to which the changes affect needs a reexecution.

Here, we compare the overhead of running ImageMagick either in a Bash script or by using Anima.

Listing 3. Bash script at run 1.

```
cd input; for e in *png; do convert $e -blur 0x5 ../out2/$e; done
cd ../out2; for e in *png; do convert $e -resize 50%x ../out3/$e; done
```

Listing 4. Bash script at run 2, after changing a parameter

```
cd input; for e in *png; do convert $e -blur 0x5 ../out2/$e; done
cd ../out2; for e in *png; do convert $e -resize 75%x ../out3/$e; done
```

Listing 5. Anima script at run 1.

```
in=INPUT(path="input")
blur=ImageMagickOperation(dir1=in, command="@in1@ -blur 0x5 @out@")
resize=ImageMagickOperation(dir1=blur, command="@in1@ -resize 50%x @out@")
```

Listing 6. Anima script at run 2, after changing a parameter.

```
in=INPUT(path="input")
blur=ImageMagickOperation(dir1=in, command="@in1@ -blur 0x5 @out@")
resize=ImageMagickOperation(dir1=blur, command="@in1@ -resize 75%x @out@")
```

The resource allocations are described in the Table 1. The Java Virtual Machine in Anduril allocates a fixed size java heap memory, but the allocation will not change with the size of the data. The second run times for Anima are clearly shorter, due to rerun prevention logic. During the second execution, Anima/Anduril will not run the blurring part at all. The time change in this small example is clear and in larger analyses a great amount of time is saved due to use of Anima.
